# A Machine Learning Model Based on Unsupervised Clustering Multihabitat to Predict the Pathological Grading of Meningiomas

**DOI:** 10.1155/2022/8955227

**Published:** 2022-09-12

**Authors:** Xinghao Wang, Jia Li, Jing Sun, Wenjuan Liu, Linkun Cai, Pengfei Zhao, Zhenghan Yang, Han Lv, Zhenchang Wang

**Affiliations:** ^1^Department of Radiology, Beijing Friendship Hospital, Capital Medical University, Beijing, China; ^2^School of Biological Science and Medical Engineering, Beihang University, No. 37 XueYuan Road, Beijing 100191, China

## Abstract

**Purpose:**

We aim to develop and validate a machine learning model by enhanced MRI to determine the pathological grading of meningiomas with unsupervised clustering image analysis method, which are multihabitat to reflect the inherent heterogeneity of tumors.

**Materials and Methods:**

A total of 120 patients with meningiomas confirmed by postoperative pathology were included in the study, including 60 patients with low-grade meningiomas (WHO grade I) and 60 patients with high-grade meningiomas (WHO grade II and WHO grade III). All patients underwent complete head enhanced magnetic resonance scans before surgery or any anti-tumor treatment. Enrolled patients in the group received surgical resection and obtained postoperative pathological data. The patients in the training group (84 people) and the test group (36 people) were randomly divided into two groups according to the ratio of 7 to 3. Multi-habitat features were extracted from MRI images based on enhanced T1. Machine learning method was used to model, which was used to distinguish high-grade meningioma from low-grade meningioma. At the same time, the obtained machine learning model was calibrated and evaluated.

**Results:**

In patients with low-grade meningioma and high-grade meningioma, we found significant differences in Silhouette coefficient (*P*<0.05). In the machine learning model, the area under the curve was 0.838 in the training group (sensitivity, 67.65%; specificity, 88.82%) and 0.73 in the test group (sensitivity, 69.05%; specificity, 71.43%). After the analysis of calibration curve and decision curve analysis, the model had shown the potential of great application value.

**Conclusions:**

Multi-habitat analysis based on enhanced MRI (T1) could accurately predict the pathological grading of meningiomas. This unsupervised image-based method could reflect the direct heterogeneity between high-grade meningiomas and low-grade meningiomas, which is of great significance for patients' treatment and prevention of recurrence.

## 1. Introduction

Meningioma is one of the most common intracranial tumors, accounting for more than one-third of all primary central nervous system tumors [1]. Meningiomas could be usually diagnosed by accident and be generally considered less malignant than other intracranial tumors [2]. Meningiomas originate from arachnoid cells located on the inner surface of the dura mater, usually from meningeal precursor cells derived from the mesoderm and neural crest. Among meningiomas, most meningiomas belong to WHO grade I benign tumors, and the recurrence rate is very low, while nearly 5% are WHO grade II/III tumors, showing a higher nature of invasion and recurrence [2, 3]. The diagnosis of meningioma largely depends on radiology. For example, imaging strongly suggests that in meningioma, biopsy is not necessary [4]. Under normal conditions, asymptomatic meningiomas grow linearly, with a growth rate of 2-4 mm/year, but there are also cases of constant volume and exponential rapid growth [5], so it is very necessary to monitor asymptomatic meningiomas. It is estimated that the 10-year overall survival rate of nonmalignant meningiomas is 81.4%, while that of grade II meningiomas is 53%, and that of grade III meningiomas is 0% [1]. The 5-year recurrence rate after total resection of grade I meningioma is 7-23%, grade II meningioma is 50-55%, and grade III meningioma is 72-78%[1, 2], which is a huge difference. At present, it is difficult to accurately judge the pathological grade of meningiomas before surgery, but it is very important for patients to choose treatment methods, formulate follow-up plans, and guide prognosis.

Magnetic resonance imaging (MRI) is widely used in the diagnosis of central nervous system diseases, including the diagnosis and detection of diseases and many other aspects. For the imaging research of meningiomas, some studies [6–8] have been carried out by using the methods of multiparameter magnetic resonance imaging and radiomics. And unsupervised clustering multihabitat [9–12] has been widely used in the evaluation of tumor heterogeneity to obtain the heterogeneity information within the tumor. Habitat analysis is an important research method gradually developed in the image field. In this study, we collected preoperative enhanced MR images of patients with meningiomas of different pathological grades for multihabitat analysis to reflect the differences in tumor heterogeneity levels among patients with meningiomas of different grades and help determine their pathological grades.

## 2. Materials and Methods

### 2.1. Patient

A total of 120 patients with meningiomas were retrospectively analyzed from May 2016 to June 2019. And all patients underwent preoperative enhanced MRI scanning and successfully obtained pathological tissue samples. Our entire experimental plan had been approved by the ethics committee, in which the images and clinical data of patients had been approved and exempted from informed consent. In the process of processing the data, considering the sensitivity and ethical requirements of the head data, we stored all the data confidentially and unlabeled it to comply with the relevant subject protection principles. The exclusion criteria were as follows: (1) patients with a history of surgery, (2) patients with a history of tumor embolism or gamma knife surgery before MRI, and (3) patients whose T1-weighted images were not clear enough to be analyzed. The patients in the training group (84 patients) and the test group (36 patients) were randomly divided into two groups according to the ratio of 7 to 3.

### 2.2. MR Imaging

All patients underwent complete enhanced MR scanning. T1-weighted, T2-weighted, and enhanced T1-weighted imaging was imaged with a 3.0 MRI system (Signa, HDxt, General Electric Healthcare, Milwaukee, WI, USA), with an 8-channel array coil. Routine sequence imaging was performed for all subjects and included axial T2WI (TR = 3520 ms, TE = 102 ms, ETL = 20, matrix size = 320 × 256) and FLAIR (TR = 8000 ms, TE = 165 ms, TI = 750 ms, matrix size = 256 × 192) and axial with coronal contrast-enhanced (CE) T1WI after injection of contrast agent.

### 2.3. Habitat Generation and Feature Extraction

**Figure 1:**
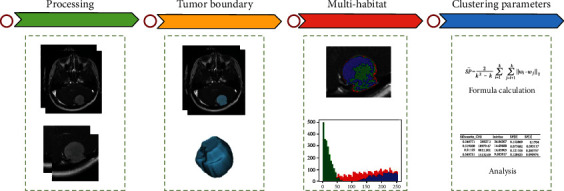
The workflow of the multihabitat.

The workflow of the multihabitat is shown in Figure 1. We selected the enhanced T1 image for processing. First, two medical imaging diagnostic doctors with more than 5 years of experience outlined the overall edge of the tumor, and the two found that it was time to introduce a third person who has more than 10 years of experience in the diagnosis of the central nervous system to judge the dispute. Although it was not a radiomics study, we refer to the Image Biomarker Standardization Initiative [13] principles for image processing methods. All tumor edge confirmation work was completed through 3D slicer software (https://www.slicer.org/). After obtaining the complete region of interest of meningioma, multihabitat [14] was implemented through self-built code in Python (3.8.5). *K*-means algorithm [15] was widely used in unsupervised image segmentation, and our heterogeneous region segmentation also adopts this kind of algorithm. In this paper, we choose a clustering parameter with *K* equal to three. We selected five clustering indicators to evaluate the tumor intrinsic heterogeneity of meningiomas: (1) inertia, within cluster sum of square error; (2) Calinski-Harabasz Index; (3) Silhouette coefficient; (4) separation; and (5) Davies-Bouldin Index.

### 2.4. Model Establishment

Through statistical judgment, we screen out meaningful clustering features for final classification. For the features of modeling, we adopt the *Z*-score method to reduce the adverse effects on the model caused by different feature distributions. For feature screening, we choose many ways of parallel or serial, such as Lasso, PCA, PCC, and analysis of variance. For the selection of machine learning classifiers, we choose many ways, including linear regression, linear regression, logistic regression, linear discriminant analysis, naive Bayes, KNN, random forest, and Gaussian regression. For the model, we used the experimental set for modeling and the verification set for verification to explore the fitting degree of the machine learning model.

### 2.5. Statistical Analysis

MedCalc version 15.2.2 (http://www.medcalc.org) and R (version 4.0.3) were used for analyses. We extracted labels from the postoperative pathological reports of patients with meningiomas, in which WHO grade I was a low-grade meningioma, and WHO grade II and grade III were a high-grade meningioma. For clustering features, first judge the data type and select the corresponding *t*-test or *U*-test. The ROC curve was used to evaluate the model, and the decision curve analysis (DCA) curve and calibration curve of the model are calculated to further describe the model. Bilateral *P* values less than 0.05 were considered statistically significant.

## 3. Results

### 3.1. Clinical Characters

We retrospectively recruited 120 patients with meningiomas, including 56 males and 64 females, 60 patients with low-grade meningiomas (grade I) and 60 patients with high-grade meningiomas (grade II and III). More information could be summarized in Table 1. There was no significant difference in clinical characteristics between the training group and the validation group (*P* > 0.05).

### 3.2. Clustering Parameters and Machine Learning Model

For all clustering parameters, we performed the operation of confusion matrix (for the label with different pathological grades). The detailed description of each parameter is shown in Table 2.

**Figure 2:**
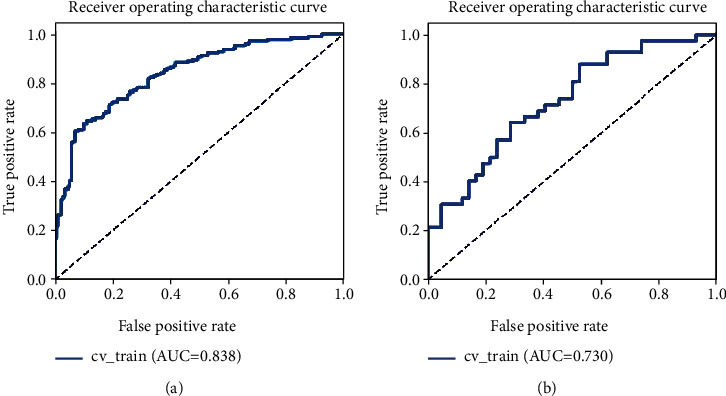
ROC of the machine learning model: (a) the performance of the training group; (b) the performance of the validation group.

**Figure 3:**
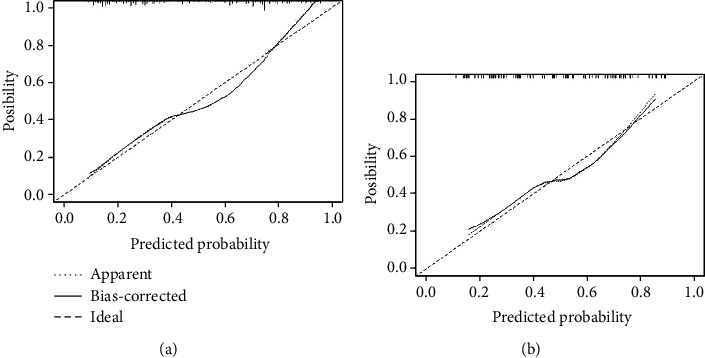
Calibration curve of the machine learning model: (a) the calibration curve of the training group; (b) the calibration curve of the validation group.

**Figure 4:**
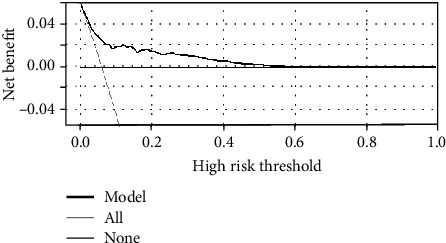
The decision curve analysis of the machine learning model.

Through the screening and dimensionality reduction of clustering features, we had successfully obtained a machine learning model based on inertia, Calinski-Harabasz Index, Silhouette coefficient, and Davies-Bouldin Index. We used Gaussian process as the classifier. Gaussian process combines the features to build a joint distribution to estimate the probability of the classification. The machine learning model had shown a great ability to distinguish different levels of meningiomas (Figure 2). We also calculated its calibration curve (Figure 3) and DCA curve (Figure 4). Through the calibration of the model, we can visually describe the fitting degree of the model. As shown in Figure 4, the decision curve of our model is all on the horizontal axis, and there is no “negative return” area, which is considered to be one of the good clinical application values.

## 4. Discussion

In this study, unsupervised clustering was used to evaluate the heterogeneity of meningiomas at different pathological levels for enhanced magnetic resonance image (T1), and a machine learning model for regression prediction was established. For the multihabitat method based on *K*-means, we have successfully divided the subregions of enhanced magnetic resonance images. Unsupervised clustering parameters were used to participate in the establishment of the machine learning model. The final machine learning model could better distinguish high-grade meningiomas from low-grade meningiomas.

Compared with low-grade (benign) meningiomas, high-grade (atypical or anaplastic) tumors have invasive biological behavior, increased risk of recurrence, and increased mortality [16]. The classification of meningioma preoperative prediction is crucial because it affects various treatment plans, including surgical resection strategies. According to the guidelines issued by the European Association of Neuro-Oncology, meningiomas found accidentally and speculated radiologically can be treated only by observation [4], so judging the pathological grade also plays an important role in the formulation of follow-up observation strategies. Due to the limitations of biopsy, in some cases, the histological grade may not be determined. At the same time, according to previous cohort studies, it is found that the correlation between clinical information such as age and gender and tumor grade is weak [16], and it is difficult to have a single clinical feature to predict the pathological grade of tumor. Previous studies [17–20] have focused on the imaging characteristics of meningiomas, diffusion and perfusion imaging, amide proton imaging, and PET to reflect the grading of meningiomas. However, there are great limitations in its grading value and application scenarios. In this paper, we found that clustering parameters and the machine learning model were related to the grading of meningiomas, which may be related to the tumor heterogeneity represented by the three signal areas of MRI, and it is also consistent with previous studies that high-grade meningiomas show more complex texture patterns [21] than low-grade meningiomas on MRI. A previous study [22] also showed that irregular tumor boundaries were associated with high-grade meningiomas, which was consistent with our results.

Medical imaging can provide full volume evaluation of the continuous nature of tumors by generating spatial resolution maps of subunits called “voxels” [23–25]. Malignant tumors have complex biology and show significant spatial variation in gene expression, biochemistry, histopathology, and macro structure. Cancer cells not only evolved from clones of single progenitor cells to more aggressive and treatment resistant cells but also showed branching evolution, so that each tumor developed and retained multiple different subclonal populations. There was a wide consensus among various theories of tumor formation that there are differences between tumor cells, progenitor cells, and cells between tumors. This genetic heterogeneity combined with spatial variation in different environments can lead to a variety of regional differences in matrix structure, oxygen consumption, energy metabolism, and growth factor expression. Therefore, with the development of different regions of the tumor, each region has spatially different blood perfusion, hypoxia, cell proliferation, apoptosis, and other characteristics [26–28]. Compared with benign meningiomas, the high-grade meningiomas [29, 30] are characterized by genomic instability, which indicates that regional changes in chromosome structure may be an important feature of recurrence, treatment resistance, and invasiveness. An innovative study [28] combining the spatial transcription information of meningiomas shows that although the MRI signals of high-grade meningiomas and low-grade meningiomas are generally the same, the signals of high-grade meningiomas are more variable. The above research shows that the MRI-based heterogeneity of meningiomas is related to the regional transcriptome differences of tumor tissues, in which the regions with high signal are rich in developmental gene expression, and these gene expression programs may be the basis of meningioma cell proliferation and tumor recurrence. Meningiomas with high proliferative potential may show a highly heterogeneous distribution of proliferating cells in tumors, and this heterogeneity may produce irregular shapes. In our experimental protocol, our unsupervised classification of red areas (high signal areas) is always located at the edge of the tumor, which is considered to be related to tumor cell proliferation and infiltration [31]. The data-driven method [14, 32–34] successfully distinguishes survival tumors from nonsurvival tumors using multiparameter MRI and verifies the method against H&E histology [35].

Different from traditional radiomics, we unsupervised the three-dimensional tumor voxels into subregions and analyzed their internal or mutual relationships to explain the spatial heterogeneity. While fully considering the internal differences of their subclassifications, clustering parameters are designed to evaluate the correlation or eccentricity of different subgroups (spatial subregions). For different parameters, they represent different perspectives to evaluate the heterogeneity components within tumors, which is undoubtedly consistent with previous studies [36–39]. In addition to the absolute characterization of tumors, we pay attention to the direct internal relations of different subregions, and their ability to respond to internal relations is stronger than direct indicators. Although many factors were considered in the study design, there were some deficiencies in this study (retrospective study). First of all, for the lack of control over information sources, one of the common problems of retrospective research is that we cannot design relevant data collection methods or other confounding factors in advance. Secondly, for the processing of brain MRI images, segmentation technology is one of the key technologies [40], but due to the limitations of experimental conditions, our preprocessing methods and segmentation methods are not accurate and automatic. Third, our research data is single center, which will undoubtedly weaken the persuasion of the results, which undoubtedly needs to be further explored in the real world of large samples and multicenters.

## 5. Conclusions

Multihabitat analysis based on *K*-means by enhanced MRI could distinguish high-grade meningioma from low-grade meningioma accurately. This unsupervised image-based method could reflect the direct heterogeneity between different grade meningiomas, which is of great importance for patients' treatment and prevention of recurrence.

## Figures and Tables

**Table 1:** The clinical characteristics of meningiomas.

Characteristic	Training group (*n* = 84)	Test group (*n* = 36)
Age (average year)	52.3	52.9
Gender		
Male	40	16
Female	44	20
PR		
Positive	39	16
Negative	45	20
Ki-67		
Positive	34	17
Negative	50	19
WHO grading		
Grade I	41	19
Grade II	34	13
Grade III	9	4

**Table 2:** Distinguishing ability of clustering parameters.

	AUC (95% CI)	Specificity	Sensitivity	Youden index
Inertia	0.597 (0.504-0.685)	46.67%	73.33%	0.20
Calinski-Harabasz Index	0.596 (0.502-0.684)	41.67%	76.67%	0.183
Silhouette coefficient	0.754 (0.668-0.828)	83.33%	56.67%	0.40
Separation	0.571 (0.477-0.661)	46.67%	71.67%	0.184
Davies-Bouldin Index	0.674 (0.583-0.757)	48.33%	80.0%	0.283

## Data Availability

The MRI images of the study are available from the corresponding authors upon request.
